# Supplementary figures and images for: α-galactosylceramide-stimulated invariant natural killer T-cells play a protective role in murine vulvovaginal candidiasis by Candida albicans
Source: PLoS One. 2021 Nov 16;16(11):e0259306. doi: 10.1371/journal.pone.0259306 (PMC8594805; doi:10.1371/journal.pone.0259306)

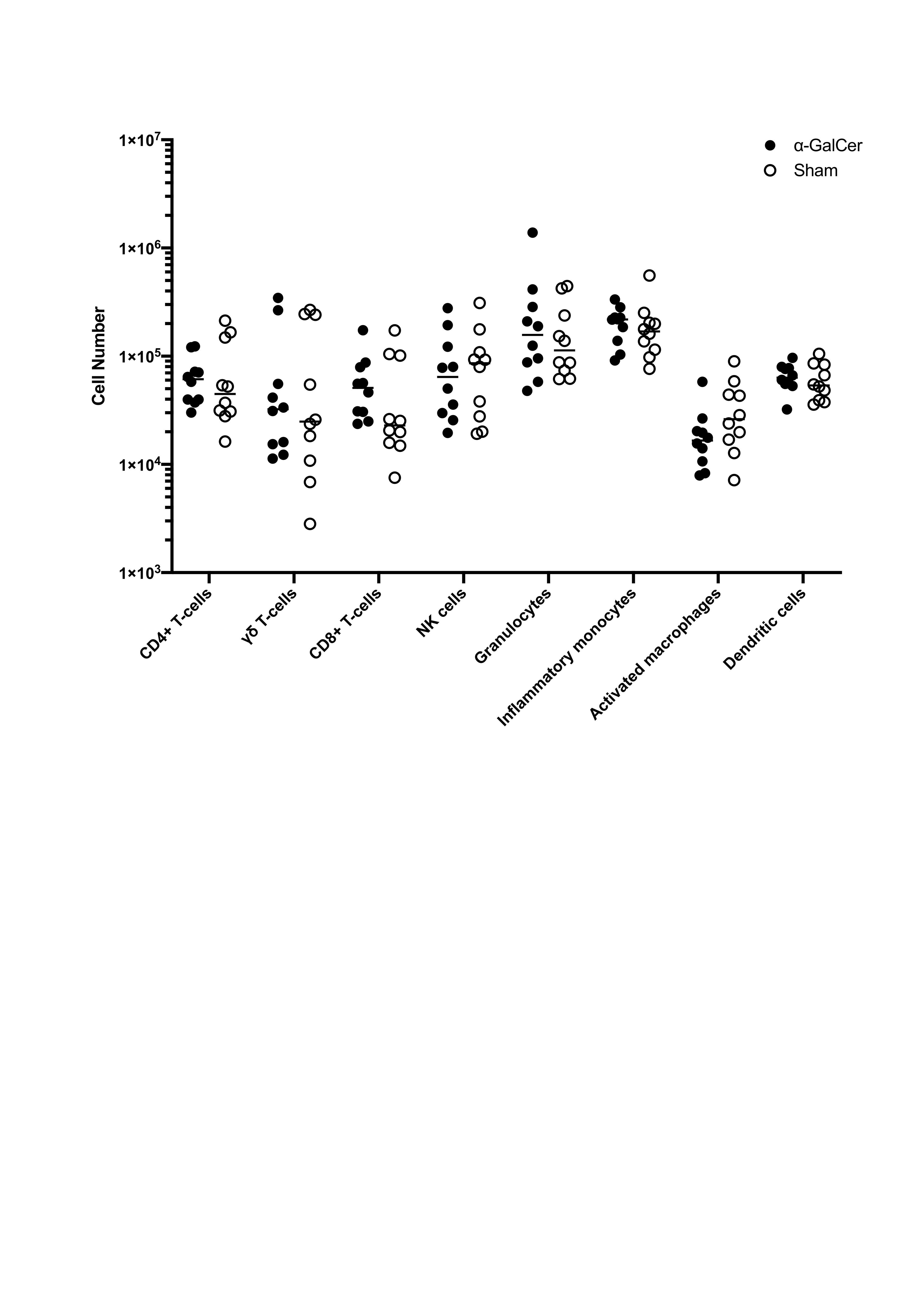

Supplement: S1 Fig — The number of uterine immune cells 3 days after C. albicans inoculation. The results of three independent experiments, each with 10 samples, were pooled for analysis. (TIF) [file pone.0259306.s001.tif]
